# Notices, Reviews, &c.

**Published:** 1859-05

**Authors:** 


### NOTICES, REVIEWS, &c.

*The Culprit Fay.* A poem, by G. J. Rodman Drake, got up in most beautiful style, by Rudd & Carlton, New York, 1859. The idea was begotten just forty years ago, on a midsummer's night, on the banks of the Hudson. It has been long pronounced by general consent to be one of the most beautiful poetic compositions in the English language.

*Two Ways to Wedlock*, by the same enterprising house, 130 Grand street. Dedicated to George P. Morris, reprinted from the rich columns of the Home Journal. The language is pure, the style flowing, the sentiments chaste and elevating, and the moral unexceptionable, while a wise and safe practical vein runs through the whole book, closing thus,—" unspoiled by prosperity, their hearts are tender and true ; and she knows that if adversity should come it will be met as it has been before, bravely and cheerfully, looking ever forward to the light beyond."

*Braithewait's Retrospect*, $2 per year ; is republished semi-annually by W. A. Townsend & Co., 377 Broadway, New York, and contains the cream of medical progress during each preceding six months. The index of the first thirty-four parts makes Braithewaite an invaluable adjunct to the library of the medical scholar.

*The Edinburgh Review* for January, $3 a year, contains articles of great interest on Life Assurance, Church Rate Question, Life and Organization, &c., all written with the accustomed ability of that long established quarterly. Leonard Scott & Co., New York.

" *Man and his Dwelling Place.*" Redfield, publisher, 391 pp. $1 25. Anonymous. This is a book of great thoughts, and for those who think. It is far reaching, and seems to be the product of a logical mind. It is styled an essay towards the interpretation of nature. Its preface is an Eastern parable on the infatuation of the greed for gold ; its last line, " It would save you to believe, to believe in Christ THE REDEEMER OF THE WORLD." The chapter on Scepticism abounds with great striking thoughts.

*Why do I Live.* Christian Association. Obedience the Life of Missions. Faith, the Principle of Missions, are four volumes abounding in hints on practical religion. A vein of deep and true and humble piety, devoted and self-denying, runs through every page ; the first by the American Tract Society, the last two by the Presbyterian Board of Education ; all written by that able and earnest man Thomas Smythe, D. D., of Charleston, S. C.

*The Evangelical Repository*,—Monthly. Philadelphia. The organ of the United Presbyterian Church of North America, abounding in safe and instructive religious reading for families. Several pages of each number show the care of that excellent people for the " little ones," the lambs of the flock, and in this is wise above many.

*Blackwood*, by Leonard Scott & Co., New York, $3 a year, maintains its old-time character.

*The Happy Home*, Boston, among other good articles, has " Lessons of the Street."

